# Supplementary material for: Valence biases in reinforcement learning shift across adolescence and modulate subsequent memory
Source: eLife. 2022 Jan 24;11:e64620. doi: 10.7554/eLife.64620 (PMC8786311; doi:10.7554/eLife.64620)
Supplement: Supplementary file 1. [file elife-64620-supp1.docx]

Appendix 1--table 1. Results from an ordinal model predicting memory performance.

| *Predictors* | *Odds Ratios* | *CI* | *p* |
| --- | --- | --- | --- |
| Linear Age | 1.03 | 0.88 – 1.22 | 0.685 |
| Quadratic Age | 0.90 | 0.74 – 1.09 | 0.286 |
| Memory Trial Number | 0.81 | 0.75 – 0.86 | **<0.001** |
| False Alarm Rate | 1.55 | 1.30 – 1.85 | **<0.001** |
| Asymmetry Index (AI) | 1.01 | 0.85 – 1.20 | 0.915 |
| PE Magnitude | 1.24 | 1.16 – 1.33 | **<0.001** |
| PE Valence | 0.91 | 0.84 – 0.98 | **0.017** |
| AI:PE Magnitude | 1.00 | 0.95 – 1.05 | 0.993 |
| AI:PE Valence | 1.10 | 1.04 – 1.17 | **0.002** |
| PE Magnitude:PE Valence | 1.00 | 0.94 – 1.06 | 0.897 |
| AI:PE Magnitude:PE Valence | 1.11 | 1.05 – 1.17 | **<0.001** |
| *Threshold Coefficients* |  |  |  |
| Definitely New\|Maybe New | 0.25 | 0.19 – 0.32 | **<0.001** |
| Maybe New\|Maybe Old | 0.83 | 0.64 – 1.07 | **0.156** |
| Maybe Old\|Definitely Old | 1.86 | 1.44 – 2.40 | **<0.001** |
